# Chinese women’s attitudes towards postpartum interventions to prevent type 2 diabetes after gestational diabetes: a semi-structured qualitative study

**DOI:** 10.1186/s12978-021-01180-1

**Published:** 2021-06-26

**Authors:** Jie Shang, Amanda Henry, Puhong Zhang, Huan Chen, Kelly Thompson, Xiaodong Wang, Na Liu, Jiani Zhang, Yan Liu, Jianbo Jin, Xiongfei Pan, Xue Yang, Jane E. Hirst

**Affiliations:** 1 School of Women’s and Children’s Health, UNSW Medicine, Sydney, Australia; 2 The George Institute for Global Health, Beijing, China; 3 The George Institute for Global Health, Sydney, Australia; 4 Department of Women’s and Children’s Health, St George Hospital, Sydney, Australia; 5 Faculty of Medicine, The University of New South Wales, Sydney, Australia; 6 Acupuncture Department, Guang’anmen Hospital, China Academy of Chinese Medical Sciences, Beijing, China; 7 Department of Obstetrics and Gynaecology, West China Second University Hospital, Sichuan University, Chengdu, China; 8 Key Laboratory of Birth Defects and Related Diseases of Women and Children, Sichuan University, Chengdu, China; 9 Antenatal Care Clinic, Shuangliu Maternal and Child Health Hospital, Chengdu, China; 10 School of Public Health, Peking University Health Science Center, Beijing, China; 11 Division of Epidemiology, Department of Medicine, Vanderbilt University Medical Centre, Nashville, USA; 12 Department of Epidemiology & Biostatistics, Tongji Medical College, Huazhong University of Science and Technology, Wuhan, China; 13 Department of Epidemiology and Biostatistics, West China School of Public Health and West China Fourth Hospital, Sichuan University, Chengdu, China; 14 Nuffield Department of Women’s & Reproductive Health, University of Oxford, Oxford, UK; 15 The George Institute for Global Health, Oxford, UK

**Keywords:** Gestational diabetes, Type 2 diabetes mellitus, Postpartum health, Qualitative research, Preventive health, Maternal health

## Abstract

**Background:**

Gestational diabetes (GDM) is a global problem affecting millions of pregnant women, including in mainland China. These women are at high risk of Type II diabetes (T2DM). Cost-effective and clinically effective interventions are needed. We aimed to explore Chinese women’s perspectives, concerns and motivations towards participation in early postpartum interventions and/or research to prevent the development of T2DM after a GDM-affected pregnancy.

**Methods:**

We conducted a qualitative study in two hospitals in Chengdu, Southwest China. Face-to-face semi-structured interviews were conducted with 20 women with recent experience of GDM: 16 postpartum women and 4 pregnant women. Women were asked about their attitudes towards postpartum screening for type 2 diabetes, lifestyle interventions, mHealth delivered interventions and pharmacologic interventions (specifically metformin). An inductive approach to analysis was used. Interviews were recorded, transcribed, and coded using NVivo 12 Pro.

**Results:**

Most women held positive attitudes towards participating in T2DM screening, and were willing to participate in postpartum interventions to prevent T2DM through lifestyle change or mHealth interventions. Women were less likely to agree to pharmacological intervention, unless they had family members with diabetes or needed medication themselves during pregnancy. We identified seven domains influencing women’s attitudes towards future interventions: (1) experiences with the health system during pregnancy; (2) living in an enabling environment; (3) the experience of T2DM in family members; (4) knowledge of diabetes and perception of risk; (5) concerns about personal and baby health; (6) feelings and emotions, and (7) lifestyle constraints. Those with more severe GDM, an enabling environment and health knowledge, and with experience of T2DM in family members expressed more favourable views of postpartum interventions and research participation to prevent T2DM after GDM. Those who perceived themselves as having mild GDM and those with time/lifestyle constraints were less likely to participate.

**Conclusions:**

Women with experiences of GDM in Chengdu are generally willing to participate in early postpartum interventions and/or research to reduce their risk of T2DM, with a preference for non-drug, mHealth based interventions, integrating lifestyle change strategies, blood glucose monitoring, postpartum recovery and mental health.

**Supplementary Information:**

The online version contains supplementary material available at 10.1186/s12978-021-01180-1.

## Plain language summary

Gestational diabetes mellitus (GDM) is a common pregnancy complication affecting up to 1 in 6 pregnant women worldwide. Whilst the condition usually resolves soon after delivery, women are at high risk of developing type 2 diabetes mellitus (T2DM). In this study, we asked women living in Chengdu, a city in western China, about what they knew about their risk of diabetes and how they felt about participating in interventions after birth to prevent T2DM.

After listening to the views of 20 women, we concluded that in this setting most women are happy to attend T2DM screening programs after birth, and would be willing to consider participating in interventions and research after birth to prevent T2DM.

The interventions most preferred were those that aimed at lifestyle changes, and many women said would like to receive this information through their smartphone, for example through an app or social media channel. Women were reluctant to take medications to prevent T2DM.

The main factors that influenced how women felt towards interventions to prevent T2DM were: (1) their experiences with the health system during pregnancy; (2) whether the home environment was supportive to make changes to diet and lifestyle; (3) any experiences of T2DM in family members; (4) their knowledge of diabetes and perception of risk; (5) concerns about personal and baby health; (6) feelings and emotions in the postnatal period, and (7) lifestyle constraints making it difficult to make dietary changes.

**Supplementary Information:**

The online version contains supplementary material available at 10.1186/s12978-021-01180-1.

## Background

Hyperglycaemia in pregnancy is estimated to affect 1 in 6 pregnant women worldwide [1]. Most of these women have gestational diabetes mellitus (GDM), and whilst this usually resolves soon after birth, women with GDM remain at greatly increased risk for the development of type 2 diabetes (T2DM) in the years following the pregnancy [1]. In China, the prevalence of T2DM has been increasing rapidly over past few decades, and is estimated to affect 10.9% of the adult population [2]. Moreover, GDM affects 14.8% of pregnant women in mainland China [3]. Given this situation, detection of GDM during pregnancy may offer an opportunity after childbirth to halt the growing burden of T2DM and its associated complications.

The US Diabetes Prevention Program demonstrated that either intensive lifestyle interventions or metformin reduces the risk of developing T2DM in those at high risk [4]. Within this landmark project, a sub-study of women with a history of GDM demonstrated a 40% reduction in development of T2DM with metformin, and 35% reduction with lifestyle interventions, at 10 years after the study [4]. However, women in the DPP were, on average, 11 years from the affected pregnancy when they enrolled in the program [4, 5]. It is recognised that many women will develop T2DM following GDM much nearer to the affected pregnancy, however to date there have not been convincing studies demonstrating the effectiveness of intervention programs commenced in the months and years after the pregnancy [6]. One of the main barriers women report to starting and sustaining lifestyle changes in the early postpartum period is competing demands on time [7]. There is a need for solutions that are acceptable to women and sustainable during the months and years immediately after birth.

The aim of this study was to develop insight into Chinese women’s perspectives, concerns and motivations towards participating in different early postpartum interventions and/or research, to prevent the development of T2DM following a pregnancy affected by GDM. We also sought to identify factors that would make participation in a postpartum intervention to prevent T2DM more or less likely in this population.

## Methods

### Study setting, including GDM diagnosis and management

This study was conducted in two hospitals, West China No. 2 Hospital (WCH), and Shuangliu Maternal and Child Healthcare Hospital (SLH), in Chengdu, the capital city of Sichuan Province in Southwest China. Chengdu represents the economic, scientific, and educational centre of the western region of China, with a permanent resident population of 15.7 million, GDP of 1120.2 billion yuan, and an urbanization rate of 71.5% [8]. WCH is the major tertiary referral centre for Chengdu and the surrounding rural population conducting approximately 20,000 deliveries per year. SLH is a district hospital in Chengdu, with approximately 3,000 deliveries per year [9]. Most women seeking care in the two hospitals are Han Chinese, with a smaller number of women from ethnic minorities including those from neighbouring Tibet.

Clinical pathways and procedures for GDM management (including screening, lifestyle advice, BG self-testing, and clinical decisions regarding insulin use) were similar in both hospitals. Briefly, all pregnant women undergo a 2-h, 75 g oral glucose tolerance test (OGTT) between 24 and 28 gestational weeks. GDM was diagnosed using International Association of Diabetes and Pregnancy Study Group (IADPSG) criteria [10]. After diagnosis, women with GDM follow a unique track from women without GDM, including receiving lectures by the nutrition department and education related to GDM. Women are advised to strictly control their diet, exercise more (e.g. walking for half an hour after each meal every day), and to purchase a glucometer to record BG results four times per day on at least two days each week. Based on these results, the obstetrician will determine whether to prescribe insulin.

### Sampling

Doctors invited pregnant and newly postpartum women by telephone call to attend a one-to-one or focus group interview by convenience sampling. Women with GDM attending regular pregnancy checks, or who had delivered their babies (within 6 months) at either of the hospitals were eligible. Doctors approached new patients until 30 women, 20 from WCH and 10 from SLH, verbally accepted the interview invitation. Ultimately, 20 women, 16 from WCH and 4 from SLH, gave written informed consent and completed the face-to-face interview. Information saturation was reached.

The study was approved by the Peking University Medical School Ethical Review Board prior to commencement. For the purposes of analysis and interpretation, any identifiable information given by participants was removed from transcripts, and each participant referred to by a unique participant number (SLxx or HXxx) to ensure confidentiality.

### Data collection

All interviews were recorded by the researchers using a mobile recording device, and were transcribed into Chinese text by a third-party commercial transcription company (Shen Shou Su Ji). A native speaker of the local accent checked the transcripts to minimize misinformation. The transcripts were then imported into NVivo 12 Pro for further processing and analysis.

### Interviews

We adopted a semi-structured interview approach to collect information on the background of the participants, their knowledge of GDM and type 2 diabetes risk, attitudes towards participation in research in the postpartum period, and specific postpartum interventions for T2DM prevention: regular screening for the development of T2DM, diet and lifestyle change programs; medications to prevent T2DM including metformin; and mHealth platforms to support interventions. We conducted individual pilot interviews with 2 participants from WCH and 1 participant from SLH in July 2019, to test the efficacy of the interview guide. Following these pilot interviews, the interview guide was unchanged. An additional file shows the interview guide in more details (see Additional file 1). The remaining interviews were conducted during September 2019 within the two hospitals.

Each interview lasted from 17 to 58 min. There were 13 women interviewed one-to-one, and 7 women interviewed in small groups of 2 or 3 participants. Individual interviews and focus group interviews were combined to increase data richness [11]. The pilot interviews were conducted by two interviewers (HC and JS), who were public health researchers from Beijing with no prior contact or relationship with any of the participants. All following interviews were conducted by the same interviewer (JS) in a private space at each hospital.

### Data analysis

Interview transcripts were imported into NVivo 12 Pro qualitative analysis software and coded in two rounds from November 2019 [12]. Inductive analysis was employed, with codes categorized based on similar characteristics by the researcher who conducted the interviews (JS) [13]. Information not aligned with the study’s research questions was removed, and categories were compared and contrasted to identify seven major domains (themes). The categorization and analysis of data were based on interview guide by the interviewer (JS) under supervision of two senior researchers with experience in qualitative research methodologies (JH and AH). Translation from Chinese to English was completed by a Masters graduate in epidemiology with 6 years study experience in an English-speaking country whose first language is Chinese (JS).

## Findings

### General attitudes towards participating in postpartum interventional research to prevent T2DM

Women expressed a range of opinions around potentially participating in postpartum T2DM preventative interventional research. While the majority of women expressed willingness to participate, some held differing views. This was related to uncertainties regarding their own T2DM risk, as well as concern about the different situations they would encounter after delivery.

"I think it is a good thing to participate in this kind of research. It can also help a lot of people. Questions like how to prevent GDM, and what can we do after GDM to control our BG, they are significant, and would benefit a lot of women. Therefore, I am happy to participate in." (HX11)

However, some women voiced concerns around the time required to attend additional visits. Participants expressed difficulties in frequently coming to the hospital due to their working status and new childcare responsibilities. Most women planned to return work after 6 months, which greatly decreases their time flexibility to participate in interventions such as regular exercise.

“I think I will (participate) if it is not too complicated or time-consuming (HX04).”

“We will be working and taking care of the child. We cannot keep coming to the hospital." (HX04-05)

Women expressed willingness to participate in interventions delivered using mHealth approaches, rather than programs requiring hospital based face-to-face sessions. Smartphone apps and WeChat functions were favoured for their time efficiency. Women felt they had benefited from the convenience of the online features of these technologies during their pregnancy, for example, making appointments, viewing hospital exam results, and information sharing.

“Something like that (WeChat microapps, pop-up messages, etc.) would be better. It would be difficult for me to come to the hospital (for postpartum interventions) frequently.” (HX07)

When asked about the different types of postpartum interventions available to prevent T2DM, there was a spectrum of opinions, with most women preferring lifestyle modifications delivered through mHealth platforms (Table 1). Women expressed positive attitudes towards postpartum lifestyle interventions, such as exercise and dietary control, which they were familiar with during pregnancy:

“I am working out nowadays myself, and I will increase the intensity (of exercise) after I stop breastfeeding. I also want to lose some weight.” (HX13)

**Table 1:** Participants’ attitudes towards T2DM prevention through postpartum interventions

Postpartum interventions	Summary of participants’ main attitudes
Regular Screening for T2DM	Ongoing screening for T2DM was acceptable to most of the women sampled (n = 18, 90%) Women felt that this would be best done at healthcare facilities, like specialized maternal and child health hospitals, and community hospitals
Lifestyle changes	Most women felt that diet and lifestyle changes would be acceptable during the postpartum period (n = 17, 85%) Women reported lifestyle interventions are in line with their goals, like losing weight Women felt they could be more flexible with lifestyle changes to suit their lives Less perception of risk to the child, especially during breastfeeding
Medications	There was a wide range of attitudes towards whether or not women would be comfortable taking medications to prevent T2DM Several women sampled stated they would refuse to participate in any drug interventions (n = 8, 40%) Safety concerns were raised around the potential harm to both the mother and the baby Women discussed that taking drugs for disease prevention is less acceptable in the cultural context of China Some women were more open to taking medications they had used for BG management during pregnancy after the birth to prevent T2DM, for example insulin and metformin (n = 4, 20%)
mHealth intervention delivery	There was a strong preference for interventions delivered through mobile platforms (n = 18, 90%) All interviewees reported using smartphone Apps /WeChat functions for maternal and child healthcare, including official Apps or WeChat functions from their hospital, as well as commercial Apps

There were a wide range of views surrounding pharmacologic agents to prevent T2DM. Women expressed concerns around taking medicines during pregnancy and particularly whilst they were still breastfeeding. Several women expressed concerns about possible safety issues and side effects, with one woman stating she would not take a medication for prevention only, and another expressing concerns about drug dependence.

“I will accept using drugs only if I had been diagnosed with DM…I don’t want to take drugs too early, which might cause (drug) dependence.” (HX06)

“I think most people may not accept drugs easily. All drugs have side effects, and may be harmful to your organs...There would be safety concerns.” (HX08)

"If I participate in a drug study, I would only agree when I stop breastfeeding. I don't want to take any drug during breastfeeding – as it is said ‘shi yao san fen du (any drug would be 30% harmful)’." (HX01)

However, a small group of women were more positive about possibly using a drug to prevent T2DM in future. These women were more likely to have had someone in the family with DM, were known to have pre-existing BG issue such as insulin resistance, had needed medication to treat their GDM, or had more background knowledge about the risk of T2DM after GDM. However, when questioned about participation in pharmacological research, they said they would only consider this if the drug was well-established, proven to be safe, or if they were familiar with it from other family members.

“I won’t accept drugs under experiment either. I would only use ‘mature’ drugs – after years of clinical use that can prove its safety.” (HX01)

“I can accept taking drugs. I know metformin – my mother is using it.” (HX03)

Metformin is not commonly used in China during pregnancy, therefore most women were not familiar with its use. One woman expressed a preference for insulin, as this was perceived by her as a more ‘natural’ alternative:

“I have concerns whether it would affect the foetus if I get pregnant again when using the drug. I think insulin is fine. I know that doctors would prescribe insulin, but not drugs. The doctor say that insulin is an existing hormone in our body, which is not harmful. Therefore, I think insulin is more acceptable.” (XH05)

Women were concerned about their postpartum BG level and generally supportive of having regular DM screening in future. Whilst most women were satisfied with having the screening test at the hospital, one woman expressed that she preferred self-monitoring her BG with remote support for professional suggestions:

"I prefer monitoring my BG at home by myself (in future). If I can get some feedback through WeChat or Apps, then it would be great." (HX02)

## Domains of GDM experience relevant to potential future research participation

Seven domains were identified that influenced women’s attitudes towards participating in postpartum interventions and/or research: (1) experiences with the health system during pregnancy; (2) living in an enabling environment; (3) the experience of T2DM in family members; (4) knowledge of diabetes and perception of risk; (5) concerns about personal and baby health; (6) feelings and emotions; and (7) breastfeeding and lifestyle constraints.

### Domain 1: Experiences with the health system during pregnancy

Lifestyle advice was offered to all women with GDM by their medical team. This advice was valued as it came from a trusted source (their doctor). Women therefore felt such changes would be acceptable to continue after birth.

Some women reported difficulty in controlling their BG during pregnancy, despite insulin or metformin. These women expressed more willingness to consider postpartum drug interventions.

“…I could not control my BG level purely by diet and exercise. Then I started to use insulin with 4 units, and then 8 units. Eventually I got 10 units. In fact. My BG level was not stabilized even after using insulin... Should I continue taking drugs? I need (professional) advice.” (HX15)

Women with other pregnancy complications, or infant health issues tended to be more aware of health risks for themselves and their offspring. They were also more likely to be willing to participate in a postpartum intervention, regardless of type.

“My child had pneumonia at around four weeks after birth. He was hospitalized for one week for treatment… I might not be able to make lifestyle changes. I would rather take drugs.” (HX09)

### Domain 2: Living in an enabling environment

Working status influenced women’s ability to manage their BG during pregnancy. Difficulties reported included limited food choices, and competing demands on their time, which they feared would remain after childbirth in addition to also facing new challenges, like breastfeeding and taking care of the newborn. For some women, medication was thought to be a less time-consuming option.

“I am a clinical doctor. I might not be able to make lifestyle changes. I would rather take drugs.” (HX09)

Family support was an important enabler to making dietary changes during pregnancy. However, for some women conflicts with other family members had made these changes much harder to achieve. Family support (or lack thereof) influenced their perceived ability to make post-partum lifestyle changes.

"My father has T2DM, so that we know what food I should choose, and how to cook. I eat same food just as my family do… After my father and I were diagnosed with DM and GDM respectively, my family has changed recipes - like eating a variety of grains rather than pure rice. My family supported me." (HX08)

In contrast:

“My family could not take good care of me regarding my GDM condition. It would be better if I live alone…My husband has been working far away, so that I had to live with my mother-in-law. She doesn’t understand my food restrictions – they contradict with her experiences." (SL02)

Some participants reported they had made connections with other pregnant women with GDM through social media chats organised by the hospital or their own social network. They had formed dynamic groups for information sharing that were sometimes moderated by hospital staff. Whilst women were positive about being part of such groups during pregnancy, the interactions had not been maintained after birth. The women interviewed expressed willingness to continue such connections in postpartum period through smartphone apps, like WeChat, and that this could help them maintain lifestyle changes.

"I have a WeChat group of this kind. We know that we all have the condition (GDM). This kind of communication usually ends after childbirth." (HX12)

### Domain 3: The experience of T2DM in family members

Five participants had experience of close family members with T2DM. Although their family members were undertaking different strategies for management, these women had greater awareness and better knowledge around diabetes. These women also expressed a greater interest in postpartum interventions for T2DM prevention.

"My grandfather has T2DM, therefore I am a bit worried. I definitely don't want to be a T2DM patient—people would suffer very much from it when approaching end of life… I would like to attend postpartum intervention if invited." (HX13).

### Domain 4: Knowledge of diabetes and perception of risk

Levels of knowledge on GDM and the sources from which women obtained this information varied. In addition to information given by doctors, women reported using online resources, consulting relatives, and friends with medical backgrounds. Women wanted more information on causes of GDM, and health outcomes. Knowledge of diabetes and perception of risk could profoundly influence women’s attitudes towards postpartum interventions:

"I know some people have higher risk (of GDM), because my mother has DM. I started to control my diet since I got pregnant…I have heard that the newborns might have pneumonia and other conditions and be hospitalized (if the mother has GDM)... Therefore I will develop DM later. I read the pop-out information (about GDM) on my mobile phone... I know metformin is a drug treating DM." (HX01)

"I did not consider myself as high-risk group (of GDM), because I was only 29 years old when I got pregnant. The critical age is 35, I was six years younger… My doctor told me that 30% of women with GDM would develop into T2DM. “(HX08)

### Domain 5: Concerns about personal and baby health

The diagnosis of GDM raised concerns for many women, including the future T2DM risk for themselves, as well as disease risk in their offspring. Women with a family history of T2DM expressed more concerns about longer-term risks, compared with women without a family history of T2DM. Some women reported that these concerns became their major motivations for BG control during pregnancy as well as after childbirth.

"… I think there should be more advocacy about the test (OGTT). I think I should have done the test even earlier. My child was born with fetal macrosomia - more than 4kg, which is due to my high BG level… My parents are both DM patients, so that I really have concern for her/his health condition in future...It would be all depending on how severe my condition is….If it gets worse, then I will accept taking oral drugs or injections if required." (HX02)

### Domain 6: Feelings and emotions

Women reported a range of emotions towards the diagnosis of GDM. Some women reported a stable mental condition throughout pregnancy, even after they have been diagnosed with GDM.

“I was not worried, because my BG is only a bit above normal. Although the doctor told me to control and monitor my BG level, I do not see it as a problem. That is because only my fasting BG value is a bit higher.” (HX10)

However, some reported more negative mental feelings, including anxiety, guilty, sadness and fright, when they found out they had GDM. Moreover, symptoms of perinatal depression were identified as an issue among some of the interviewees.

"I was nervous and sad after diagnosis. My mood was unstable at beginning of pregnancy, because it was not easy for me to get pregnant. When it stepped forward into the third trimester, I thought it was getting better. However, the GDM diagnosis and its bad influence on the offspring made me very frustrated... Since I started changing my lifestyle to control my BG, I became quite agitated and sensitive.” (SL02)

Some women spoke of serious emotional disturbances and distress during the “Zuo Yue Zi” (doing-the-month) postpartum period. This Chinese tradition involves minimal physical activity and altered dietary habits of postpartum women for at least one month after childbirth, which might include lack of fresh vegetables and alcohol-containing soup (rice wine) intake [14]. For some, there were prior mental health issues, as well as challenges with role changing of becoming a new mother, and tensions between the women and their family members. Anxiety and depression could be a barrier for early engagement with postpartum interventions for T2DM prevention.

“Actually it (depression) started just after delivery, when I was attending the 'Yuezi Centre' (a type of commercial organization for early parenting support). There were some signs and reasons. It is indeed associated with your family. There were contradictions about views and things between you and your family that affected you both physically and mentally. However, I think it depends on yourself to overcome the condition. I 'walked out' (from the depression mood)." (HX13)

### Domain 7: Breastfeeding and lifestyle constraints

Breastfeeding and taking care of the infant meant there was limited time for lifestyle change maintenance. This can influence women’s willingness to participate in postpartum interventions.

"I controlled my BG well during pregnancy. However, after delivery, I returned to my usual eating habit (not controlling diet anymore)." (HX04)

Breastfeeding women have special recipes and complex food restrictions, especially for “Zuo Yue Zi” (doing-the-month period), according to Chinese tradition and culture. Medication during this period is also highly sensitive. It is therefore rather difficult for women to continue the lifestyle advice they had started during pregnancy.

"If I can choose the time starting the intervention, I will do it at least 6 months postpartum (after breastfeeding stops). I have heard that exercising or working out would change (something of) your breastmilk." (SL03)

However, some women took actions to maintain the lifestyle changes for T2DM prevention.

“…. I started exercising immediately after 'Zuo Yue Zi'. I do not want to be diagnosed with T2DM. There are so many critical complications after T2DM. I am very clear about that.” (HX08)

## Discussion

This study aimed to lay the foundation for future interventional research to prevent T2DM after a pregnancy affected by GDM in China. Our findings indicate that the majority of women with GDM are willing to participate in interventions, including regular screening for T2DM, with a preference for lifestyle interventions delivered through mHealth platforms. Whilst most women were hesitant to use or participate in research with metformin or another pharmacologic agent as a preventative agent, those who had experienced more severe GDM, or who with a family member with T2DM were more willing to consider pharmacologic preventative strategies.

Findings in this study broadly agree with a previous systematic review of qualitative studies conducted in other countries which investigated the opinions and perspectives of women towards postpartum healthcare seeking after GDM [15]. Similar to findings of this review, the changing role of becoming a mother, including tiredness and competing demands on their time, are consistently reported as major barriers to healthcare seeking for women postpartum. Some women in our study expressed that conforming to lifestyle change interventions could be difficult, because of lack of time for regular exercise and dieting after delivery. Other issues, including a lack of any specialized postpartum care clinics, difficulties in appointment scheduling with healthcare providers, and poor family support, can all contribute to unsatisfactory compliance with prevention programs, as found both in our study and previous research [16–18]. Postpartum women in China also have concerns regarding working status. In China, maternity leave is limited to 6 months [19]. Some of the women in our study expressed that whilst they wished to continue to breastfeed after their maternity leave, this could result in further difficulties finding time for lifestyle change. Breastfeeding is one of the few interventions with evidence for positive beneficial effect on prevention of T2DM after GDM [20]. Lifestyle interventions aimed at women in the postpartum period should incorporate breast feeding support along with support for healthy eating and exercise.

We present evidence that women in western China face similar barriers to healthcare seeking after GDM and the importance of individual perceptions of future T2DM risk [15, 21]. Elevated awareness of T2DM risk after GDM in this population could facilitate participating in a postpartum program and this study provides further support for the importance of targeted health education for women to reduce the risk of T2DM.

Whilst previous research in this area has identified financial barriers towards participation in prevention programs for T2DM after GDM, like insurance coverage, and out-of-pocket expenditure, we did not identified this as a key factor in this study [15]. This is likely be due to differences in health financing models and out of pocket expenditure on health in Europe, North America and Australia. In China, GDM, and other related pregnancy and obstetric complications, are fully covered by insurance schemes [22]. The social health insurance scheme in China, including both urban and rural medical insurance packages covering nearly its whole national population, has been continuingly consolidated, and the proportion of out-of-pocket expenditure is largely decreased in recent years [22].

Women were keen to use mHealth based interventions for postpartum support. In our study nearly all interviewees reported positive experiences using smartphone Apps (or WeChat functions) during the pregnancy. The development of such apps has been very rapid in China, with thousands of apps now available for pregnant women [23]. Women in the study described use Apps to communicate with peers and health professionals, booking hospital appointments, and joining online groups through WeChat functions which provided support. Use of apps also allows greater time flexibility compared to face to face appointments. In addition, mHealth based technologies can be culturally tailored to better assist T2DM prevention after GDM [16, 24]. For instance, the tradition of ‘Zuo Yue Zi’, (“doing the month”), greatly decreases physical activity and disturbs normal dietary habits (e.g. use of alcohol-containing soup, lack of fresh vegetables) of postpartum women for at least one month after childbirth [14]. MHealth could be an effective tool to support women continue lifestyle changes even during this month, for instance, suggesting exercises that can be done indoors, and ensure when the month ends they are supported to resume healthy lifestyle choices.

### Strengths and limitations

This study is the first qualitative study from Southwest China to explore the views and perspectives towards postpartum interventions for T2DM prevention in women with GDM. Whilst invitation to the study was done by doctors not involved in this research, the study sample from both urban and rural population was generally representative of women with GDM in this region. Moreover, the sample size was sufficient to provide saturation of themes [25]. However, fewer women were recruited from SLH, and all these women were interviewed during prenatal period. Participants from SLH were less likely to accept the invitation to go back to the hospital several months after delivery. The lack of comparative data from postpartum women in rural area is a limitation of this study. The failure to recruit rural postpartum women to this study may be indicative of challenges these women face in attending hospital-based postpartum interventions. Comparatively, participants from WCH were interviewed on the day of the 6-month postpartum free OGTT check, which is well attended by urban women.

### Conclusion

We found that women diagnosed with GDM in Chengdu were generally willing to participate in interventions and research to decrease their risk of developing T2DM. There was a preference for postpartum lifestyle modifications. Pharmacological interventions for prevention of T2DM had less support, particularly amongst those who perceived themselves as having ‘mild’ GDM. We identified seven domains influencing women’s attitudes towards participation in postpartum interventions. In this population, mHealth approaches are likely to be an effective way to deliver health promotional advice to newly postpartum women, although how effective these methods will be in achieving sustained lifestyle change and health benefits needs to be evaluated. Other potential strategies to maximise participation include delivery of programs close to home and culturally sensitive interventions. Pregnancy offers a unique opportunity to prevent type 2 diabetes in women, however it is unlikely that one postpartum intervention strategy will be effective or acceptable to all. The challenge is developing programs to provide individualised support for postpartum women, equitably and affordably at scale.

## Supplementary Information

**Additional file 1.** Interview guide.

## Data Availability

The datasets during and/or analysed during the current study available from the corresponding author on reasonable request.

## Abbreviations

IADPSG: International Association of Diabetes and Pregnancy Study Group
T2DM: Type 2 diabetes mellitus
GDM: Gestational diabetes
WCH: West China No. 2 Hospital
SLH: Shuangliu Maternal and Child Healthcare Hospital
OGTT: Oral glucose tolerance test
