# Supplementary material for: Chinese women’s attitudes towards postpartum interventions to prevent type 2 diabetes after gestational diabetes: a semi-structured qualitative study
Source: Reprod Health. 2021 Jun 26;18:133. doi: 10.1186/s12978-021-01180-1 (PMC8236134; doi:10.1186/s12978-021-01180-1)
Supplement: Supplementary file 1 — Additional file 1. Interview guide. [file 12978_2021_1180_MOESM1_ESM.docx]

Interview guide

The purpose of this interview is to explore the knowledge and attitudes of women with a history of gestational diabetes, as well as their understanding of the impact of gestational diabetes on future health. Most importantly, during interviews, you need to encourage women to tell their stories and ask questions in a way that stimulates dialogue, not just asking questions that can be answered with "yes" or "no."

The interview process will be recorded to ensure that the participants’ answers are confidential and that their identities cannot be identified during data analysis. Before the interview starts, each participating woman needs to sign an informed consent form to ensure that they agree to participate in the discussion process and be recorded.

When starting a group discussion, you should ask some questions that can relax the participants and encourage them to discuss.

**Part 1. Welcome everyone to participate in this panel discussion. The purpose of today's discussion is to gain insights into the conditions of women who have had gestational diabetes during pregnancy, and to understand whether you think that gestational diabetes will still affect your health and your baby's health (risk) after delivery.**

*Before starting the discussion, we'd better introduce ourselves, tell everyone the age of the baby, and what do you think is the best and worst part of being a new mother.*

*Thank you all. Next, we will discuss some experiences and knowledge about gestational diabetes.*

What is it like to have an OGTT test during pregnancy?

• Are there any side effects?

• Do you think that some women are at greater risk of developing gestational diabetes?

• Who is more at risk?

• How did you find out that you have gestational diabetes?

• How does it feel to have gestational diabetes?

• What are you most worried about after getting gestational diabetes?

• After you got sick, what advice did your doctor give you?

• Do you find these suggestions useful?

• After discovering that you have gestational diabetes, what changes have you made in your diet or lifestyle?

• Can you follow the doctor’s advice?

• What difficulties did you encounter?

• What methods/advices would help you?

• Do you need to monitor your fingertip blood glucose during pregnancy?

• How often?

• What do you think is the purpose of measuring fingertip blood glucose?

• Does the doctor recommend that you take/inject drugs to treat gestational diabetes?

• Are these drugs troublesome to take/inject?

• Are there any side effects?

• After childbirth, do you understand the future health risks/impacts of gestational diabetes for you or your baby?

• Do you think that due to your history of gestational diabetes, you are at increased risk of other diseases? Do you know what these diseases include?

• Are there any precautions?

**Part 2. Women with a history of gestational diabetes have an increased risk of type 2 diabetes.**

• Have you heard of this risk before?

• Have you heard of any traditional medicines or measures that can prevent type 2 diabetes?

• How worried are you about developing type 2 diabetes?

• Do you think you are in a high-risk state?

• Do you know people with type 2 diabetes?

• Do you know how type 2 diabetes affects your health?

**Part 3. We are developing some measures to prevent women who have had gestational diabetes from developing type 2 diabetes within a few years after delivery. This will involve changes in diet and exercise, or a drug called metformin. The drug has been proven in international studies to effectively prevent type 2 diabetes.**

• In your opinion, what are the main difficulties for new mothers participating in diet and exercise programs?

• Are there any measures to solve these problems?

• Do you use a smartphone?

• What do you think about using APP to promote diet management and exercise?

• Are you still breastfeeding? /How long did you breastfeed after childbirth?

• Are you willing to take medication during breastfeeding to prevent type 2 diabetes? If there is drug has been proven to be safe. What are your concerns?

• Have you heard of the drug metformin before?

Have you heard of randomized trials before? Do you know what a randomized trial is like?

• If while participating in a study, you will be randomly assigned to one of the two treatment options and cannot choose by yourself, what would you think? (Is it acceptable? Why?)

• What do you think is the most convenient place for a new mother to participate in the type 2 diabetes screening?

• Are you interested in learning more about the project?

Thank you for participating in this group meeting. Do you have anything more to add?

(end)
